# Surface-enhanced Raman spectroscopy investigation on human breast cancer cells

**DOI:** 10.1186/1752-153X-7-37

**Published:** 2013-02-25

**Authors:** Jichun Zhu, Jing Zhou, Jianyu Guo, Weiying Cai, Bo Liu, Zugeng Wang, Zhenrong Sun

**Affiliations:** 1 State Key Laboratory of Precision Spectroscopy (East China Normal University), and Department of Physics, Shanghai 200062, P. R. China; 2 Department of Physics and Electronics, Henan University, 475004, Kaifeng, P. R. China

**Keywords:** SERS, Breast cancer cell, Gold nanoparticles, SERS mapping

## Abstract

**Background**

Nanoparticles are potentially used for early cancer detection, accurate diagnosis, and cancer treatment.

**Results**

In this paper, the breast cancer cells treated with gold colloidal suspension were carefully studied by surface-enhanced Raman scattering (SERS) spectra. Raman spectroscopy combining with high-resolution electron microscope is employed to investigate the interaction of gold nanoparticles (GNPs) with the intracellular components. The TEM images show that the GNPs are taken into the living cells and enveloped into some vesicles named ‘lick up vesicles’ in the cytosol.

**Conclusions**

The SERS spectra and SERS mapping of cells indicate that the major Raman bands are mostly assigned to the vibration characteristics of proteins, and the C-H in-plane bending mode of the substituted benzene in Phenylalanine is remarkably enhanced. Finally, the interaction mechanisms of the GNPs with the intracellular components are further discussed in detail.

## Introduction

Raman spectroscopy, as a nondestructive spectral technique, provides large information on the chemical structure of the probed substances, and therefore usually served as a well-established tool for investigating the complex biological systems. Since the pioneering work on the confocal Raman microscopy of the living cells and chromosomes by Puppels et al. [1], Raman spectra have been widely employed to investigate the living cells, and many significative results have been achieved [2-6].

In surface-enhanced Raman scattering (SERS), the Raman signals can be enhanced by many orders of magnitude when the probed molecules are attached or in very close vicinity to noble metal nanostructures with their high local optical fields [7]. SERS spectroscopy can provide the local chemical composition of biomolecules at very low concentrations, and detect the slight changes of the structure at sub-cellular level. In addition, SERS spectroscopy can be applied in physiological-like conditions without label and fixation. In the past twenty years, SERS spectra have been successfully performed on investigating the interaction of the nanoparticles with the living cells, such as single-molecule detection of cellular proteins, the conformational transformation, and the structural change [8-11]. Since the original work by Kneipp [12] et al., the gold nanoparticles (GNPs) have been widely used as the SERS active substrate to enhance the Raman signals for their favorable physical and chemical inactivity properties and biocompatibility [13,14]. Previous studies showed that the main route for the GNPs entrance into the living cells was through endocytosis [15-17]. However, the location and the interaction mechanisms of these GNPs with the complex intracellular microenvironment are still unclearly by now. So, it is necessary and significative to explore the interaction mechanisms and the binding sites of these GNPs with intracellular components. Previous research [18] studied the interactions between gold nanoparticles and biomolecules. In this paper, the SERS spectra and their mapping image of the native chemical components of the MDA MB 231 cell have been detected, and high-resolution electron microscopy are employed to observe the GNPs in the living cells. Furthermore, the interaction mechanisms of these GNPs with intracellular components are discussed and analyzed. This works may be benefit to further study of anticancer drug targets therapy [19,20].

## Experimental

### Gold nanoparticles (GNPs) synthesis

GNPs were synthesized by citrate reduction of HAuCl_4_, as previously described [21], and characterized by absorption spectroscopy and transmission electron microscopy. Briefly, 10mg sample of HAuCl_4_ was suspended in the 100mL distilled water, and rapidly heated to 100°C. Then 1.0mL 1% sodium citrate solution was quickly added into the boiling solution, and the solution was kept boiling for 15min.

### Cell culture and nanoparticle co-incubation

Human breast cancer cells MDA MB 231 were presented from Prof. Lai (East China Normal University). The cells were taken out from 80°C freezing icebox and thawed, washed, then incubated in glucose Dulbecco’s modified Eagle’s medium (DMEM) (Invitrogen, Carlsbad, USA) with the 10% fetal bovine serum (FBS) and the 1% penicillin/streptomycin at 37°C in the 5% CO_2_ humidified atmosphere. After twice inoculations, the cells were transferred to a 6-well cultivation plate. Three wells of cells were used as the control group without the treatment and the others were treated with the gold colloid. Then they were incubated at 37°C for 24h. Before measurements, the cells were digested with the 0.25% trypsin solution and suspended in culture media (DMEM). The collected cell suspensions were washed thoroughly with the phosphate buffered saline (PBS) and centrifuged twice at 3000 rpm for 5min to remove the culture media. The cells were suspended in the PBS buffer throughout the experiments. For the SERS mapping experiments, the cells incubated with GNPs were seeded on the quartz slide in DMEM for 24h. Before Raman experiments, the cells were rinsed with PBS and fixed by 4% paraformaldehyde at 4°C for 2 h and stored in PBS.

### Raman spectra and their mapping images

Raman spectra and Raman maps were acquired using a confocal micro-Raman spectrometer (Jobin-Yvon, T64000) equipped with a 785 nm diode laser and an integrated Olympus IX81 microscope. The Rayleigh radiation was blocked by a holographic notch filter, and the back-scattered Raman light was dispersed by a holographic grating (600 grooves/mm) onto a liquid-nitrogen-cooled CCD chip consisting of an array of 1,024 × 256 pixels. The frequency calibration was set by reference to the 520 cm^−1^ vibrational band of a silicon wafer. The laser power at the sample was approximately 3 mW to ensure no sample degradation. A 60× microscope water immersion objective with the laser spot size of about 1μm was used to focus laser and collect the Raman scattering from the individual living cell suspended in PBS buffer. SERS spectra in the range of 600 ~ 1700 cm^-1^ were recorded with a collection time of 1s. Over 200 spectra from 30 different cells were evaluated, and at least 5 spectra were acquired from each cell. Raman mapping experiments were achieved with a computer-controlled *x*,*y*-stage from a fixed single cell by using a 100× microscope objective (NA 0.95, Olympus) with the 1 μm step. Raman mapping images were constructed by collecting Raman spectra over the previously defined range of 10 × 10 μm^2^ with a collection time of 5s for one mapping spot. Spectral acquisition and spectral analysis were carried out by the Labspec6.0 software. The display scheme chosen for the image maps shows a range of SERS signal intensities on a rainbow-colored continuum: from black (little or no signal intensity) through blue, green, indigo, red, pink and yellow (highest signal intensity).

### Other instruments

The UV–VIS spectra were measured by Varian Cary 100 Spectrometer (Varian, America), and the high-resolution image of breast cancer cell was performed by Transmission Electron Microscope (JEM-2100, Japan).

## Results and discussion

**Figure 1:**
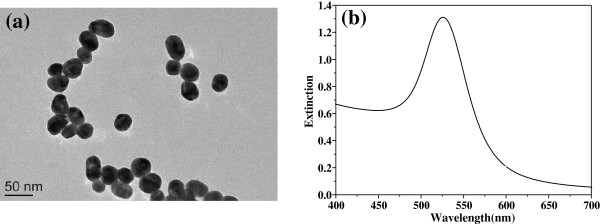
**Transmission electron micrograph image of the GNPs (a) and the extinction spectrum of gold colloids (b).**

**Figure 2:**
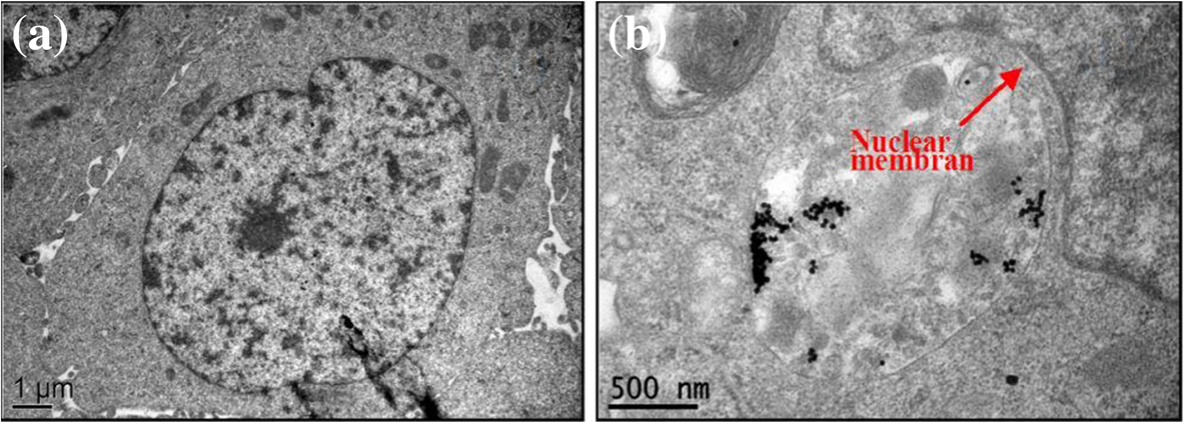
**Transmission electron micrograph images of the breast cancer cell (a) and incubated with GNPs (b).**

Figure 1 shows the transmission electron micrographs of the GNPs (a) and the UV-visible absorption spectra of the gold colloids (b). It shows that most of the GNPs are almost spheroid with the average diameter of about 35nm and the maximal absorption of the gold colloids is at 520nm. Figure 2 shows the transmission electron micrographs of the breast cancer cells (a) and that incubated with GNPs (b), respectively. It can be seen that the human breast cancer cells are approximately10μm in diameter, and the cell structures can be distinctly distinguished (e.g., nucleus, nuclear membrane).

As shown in Figure 2b, the GNPs reside in cytoplasm and are enveloped into some vesicles (named ‘lick up vesicles’), and no GNPs have been observed to enter into the nucleus. In general, these vesicles are considered as the self-protection structure to refuse the GNPs rambling in the cell. Interestingly, the GNPs have an obvious tendency to aggregate into clusters, and it results in the Raman enhancement and fluorescence suppression for closely associated molecules [22].

**Figure 3:**
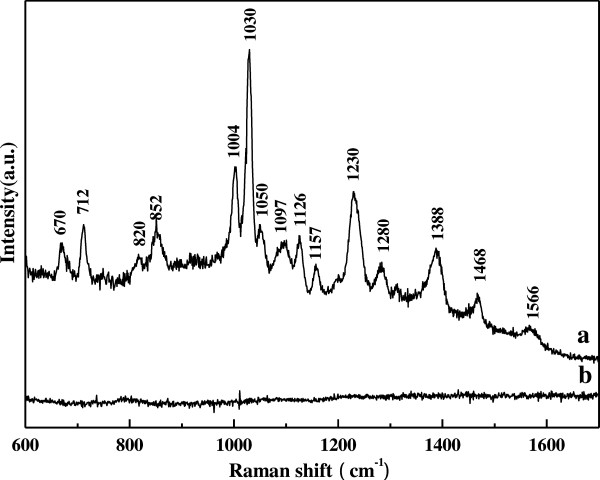
**The SERS spectrum (a) and normal Raman spectrum (b) of breast cancer cell.**

Figure 3 shows the typical SERS spectra measured from a single 231 cell after incubated with the gold colloids (a) and the normal Raman spectra (b). The SERS spectra show high signal-to-noise ratio whereas the normal spectra are not observed at the same experimental conditions. The SERS spectra are collected from the different sections of the cell and then averaged. Our experiments show that the 35nm GNPs do not influence the viability of the cells and also provide a strong SERS factor. The detailed Raman band assignments [5,6,17,23-26] in Figure 3 are presented in Table 1. The major Raman bands in Figure 3 can be mostly assigned to proteins, and the strong Raman bands at 1004, 1030, 1230 and 1388 cm^−1^ are assigned to the symmetric ring breathing mode of phenylalanine, the C-H in-plane bending mode of the substituted benzene in phenylalanine, the Amide III and the C-H stretching vibration, respectively. The relative weak Raman signals at 820 and 852 cm^−1^ are due to the Fermi resonance between the ring breathing vibration and the overtone of an out-of-plane ring bending vibration of the tyrosine parasubstituted benzene. In addition, the rather weak Raman signal at 1126 cm^-1^ can be assigned to the C-C the stretching mode of proteins.

**Table 1:** Assignment of SERS spectra measured from breast cancer cells

**Bands (cm**^**-1**^**)**	**Tentative assignments**	**Molecular origin **[5,6,17,23-26]
672	C-S str.	proteins
712	C-N str.	lipids
820	tyr	proteins
852	tyr	proteins
1004	Phe	proteins
1030	Phe	proteins
1053	C-C str.	lipids
1097	PO_2_^-^	Nucleic acids
1126	C-N, C-C str.	proteins
1157	C-C, C-N str.	proteins
1230	Amide III	proteins
1280	CH twist and bend	proteins
1388	C-H str.	proteins
1471	C–H def./bending	proteins
1566	Amide II	proteins

Abbreviations: Phe, Phenylalanine; Tyr, tyrosine; def, deformation; str., stretching.

In order to explore further the interaction mechanisms of the interaction between the gold clusters and intracellular components, the Raman band at 1030 cm^−1^, assigned to the C-H in-plane bending mode of the substituted benzene in phenylalanine, is highlighted and extremely enhanced. As well known, SERS has a selective effect for those molecules or molecular groups in vicinity of the colloidal gold clusters [8,27]. According to Ref. [28], the vibrational mode of the adsorbed molecule has a large polarization component perpendicular to the nanoparticles surfaces, and it results in the efficient enhancement of their Raman bands. So, the experimental results suggest that there should be strong affinity between GNPs and the substituted benzene of phenylalanine. The previous study [29] indicated GNPs could be in contact with mitochondria, whereas the binding sites were not analyzed. In general, phenylalanine can adsorb on the surfaces of the GNPs, and it results in enhancing the C-H in-plane bending mode of the substituted benzene in phenylalanine. Of course, there must be more other relative weak binding sites of intracellular components on GNPs, and it results in their Raman signal enhancement. Here, these binding sites are not discussed one by one.

**Figure 4:**
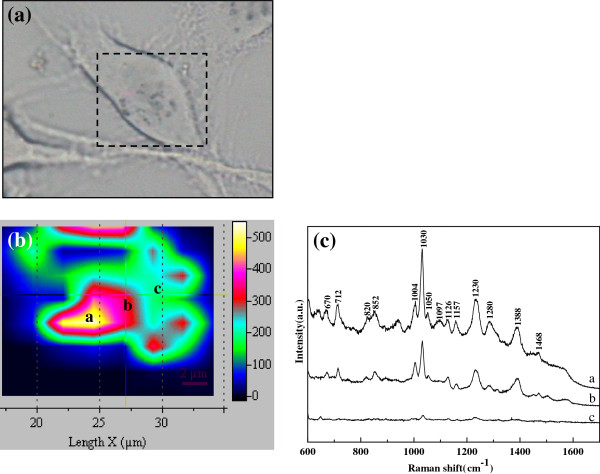
(**a**) **Photomicrograph of a fixed breast cancer cell.** (**b**) **SERS mapping image at the 1030 cm^-1^ Raman band collected in the rectangle (10 × 10 μm^2^ dimension) shown in** (**a**). (**c**) **The corresponding SERS spectra of the spots a, b and c in** (**b**).

In order to overcome the instability of the living cell in PBS, SERS spectroscopic imaging has been performed on a fixed cell incubated gold colloids. The SERS mapping approach can record a full SERS spectrum over a two-dimensional (2D) region of a cellular sample, which results in an “image map” that shows the location and distribution of the GNPS from their SERS signal intensity at that particular band. Figure 4a presents the photomicrograph of a fixed breast cancer cell, and the SERS mapping image is obtained in the labeled rectangle region. The intensity of a particular Raman band (e.g., 1030 cm^-1^) in the spectrum can be plotted using a color gradient or a series of contours. Figure 4b shows the SERS mapping image at 1030 cm^-1^ (the C-H in-plane bending mode of phenylalanine) over the 10 × 10 μm^2^ area of the cell. Figure 4c shows the corresponding SERS spectra of the spots a, b and c in Figure 4b and their features are very similar to that in Figure 3. The remarkably enhanced Raman band at 1030 cm^-1^ can be assigned to the C-H in-plane bending mode of the substituted benzene in phenylalanine, and the significant differences in the Raman intensity are related to the distribution of GNPs in the cellular microenvironments.

Generally, phenylalanine should be mainly present in the cytoplasm [12], and the GNPs also reside in cytoplasm as described in this study. The maximum signal of the Raman band appears near the cytoplasm, which indicates that there should be strong affinity between GNPs and phenylalanine. It can be also deduced that some groups of phenylalanine are exposed easily to the gold nanoparticles surface after cells incubated with the gold colloids, which results in the Raman signal enhancement. The results will be benefit to further study of anticancer drug targets therapy.

## Conclusions

In summary, in this paper the SERS spectra for the breast cancer cells incubated with GNPs have been explored. The results show the GNPs are taken into the living cell and enveloped into some vesicles named ‘lick up vesicles’ in the cytoplasm whereas not into the nucleus. The SERS spectra and its mapping image at 1030 cm^-1^ (the C-H in-plane bending mode of the substituted benzene in phenylalanine) shows that the GNPs mainly interact with protein through the binding site of phenylalanine. The experimental results indicate that SERS can be used to obtain molecular level information from cells, and has potential application in medicine and biotechnology.

## Competing interests

The authors declare that they have no competing interests.

## Authors’ contributions

JCZ performed the experiments, analyzed the data and wrote the paper. JYG and JZ performed the experiments. WYC and ZGW analyzed the data. BL revised part of the manuscript and gave final approval of the version to be published. ZRS planned the experiments, co-wrote the paper and gave final approval of the version to be published. All authors read and approve the final version.
